# Gut microbiota associations with metabolic syndrome and relevance of its study in pediatric subjects

**DOI:** 10.1080/19490976.2021.1960135

**Published:** 2021-09-07

**Authors:** Ana K. Carrizales-Sánchez, Tomás García-Cayuela, Carmen Hernández-Brenes, Carolina Senés-Guerrero

**Affiliations:** a Tecnologico de Monterrey, Escuela de Ingenieria y Ciencias, Zapopan, Jalisco, Mexico; b Tecnologico de Monterrey, Escuela de Ingenieria y Ciencias, Monterrey, Nuevo Leon, Mexico

**Keywords:** Gut microbiota, metabolic syndrome, microbiome, microbial metabolites, pediatric subjects, school-age children

## Abstract

Childhood obesity and T2DM have shown a recent alarming increase due to important changes in global lifestyle and dietary habits, highlighting the need for urgent and novel solutions to improve global public health. Gut microbiota has been shown to be relevant in human health and its dysbiosis has been associated with MetS, a health condition linked to the onset of relevant diseases including T2DM. Even though there have been recent improvements in the understanding of gut microbiota–host interactions, pediatric gut microbiota has been poorly studied compared to adults. This review provides an overview of MetS and its relevance in school-age children, discusses gut microbiota and its possible association with this metabolic condition including relevant emerging gut microbiome-based interventions for its prevention and treatment, and outlines future challenges and perspectives in preventing microbiota dysbiosis from the early stages of life.

## Introduction

Metabolic syndrome (MetS) has gained global relevance since it has shown to be strongly related to the onset of important maladies such as type-2 diabetes mellitus (T2DM) and cardiovascular diseases (CVD) and, nowadays, its prevalence in children and adolescents has been alarmingly increasing.^1^ This metabolic condition is reversible and even preventable; therefore, early diagnosis and treatment are necessary to avoid future serious health complications and the premature onset of diseases. Recently, microbiome-based interventions are gaining popularity to treat and prevent metabolic disorders, as studies in mice suggest that there is a possible relationship between the gut microbiome and MetS.^2^ Furthermore, research in humans has revealed discoveries regarding gut microbiota-host interactions, but most of it has been mainly conducted in adults due to constraints in ethical and practical considerations or even difficulties in acquiring samples from pediatric subjects, even though the gut microbiota of children have been shown to be more flexible to changes derived from environmental factors (i.e. diet) compared to adults, despite it being previously believed that the stabilization of gut microbial diversity occurred after the age of 3 years.^3^

## Metabolic syndrome and relevance of its study in pediatric subjects

Global childhood obesity had a dramatic 10-fold increase in the last four decades among children from 5 to 19 years old irrespective of socioeconomic status.^4^ This is relevant since previous statistical studies in the Danish population have significantly correlated high body mass index (BMI) values in individuals from 7 years old to early adulthood with an increased risk of developing adult T2DM if this condition is maintained until puberty or later.^5^ Moreover, it is predicted that 6–39% of pediatric subjects with obesity may also be suffering of MetS, presenting fluctuations in its prevalence depending on the applied diagnostic criteria since there are no standardized international criteria to diagnose this metabolic condition in children and adolescents.^6^

MetS, also known as “Syndrome X”, is an acquired condition characterized by possessing at least three of the following cardio-metabolic abnormalities: high blood pressure, increased central adiposity, hyperglycemia, high triglyceride levels, and decreased high-density lipoprotein cholesterol (HDL-c) levels.^7^ Its pathophysiology is a result of genetic predisposition, demographic background, and lifestyle factors such as sedentarism and diets rich in refined carbohydrates, sugars and fats.^8,9^ Notably, a relationship between gut microbiota and MetS was published a decade ago, where 16S rRNA sequencing determined that Toll-like receptor (TLR) 5 knock-out mice (T5KO) had gut microbial alterations linked with increased low-grade proinflammatory signaling that ended in MetS, validating it with gut microbiota transplantation from T5KO into wild-type (WT) germ-free mice, where all of the key features of MetS were developed after the procedure.^2^

Bacteria are the most predominant microorganisms that inhabit the gut. Firmicutes, Bacteroidetes, Actinobacteria, Fusobacteria, Proteobacteria, and Verrucomicrobia are the most abundant phyla in humans, with 90% corresponding to Firmicutes and Bacteroidetes.^10^ Moreover, the gut microbiota is shaped by multiple factors such as diet, drug treatments, and geographic region.^11–13^ Previously, host genetics was believed to affect gut microbiota, but this remains unclear as no significant associations have been reported, suggesting that it may depend more on environmental factors.^14^

Aging also seems to affect gut microbial communities. Studies in healthy Japanese subjects from 0 to 104 years old helped to classify them into two clusters: “adult-enriched” and “infant/elderly-enriched” gut microbiota. In children <20 years old, studies showed that the gut microbial maturation process was still taking place since alpha-diversity and abundance groups of some genera belonging to *Lachnospiraceae, Bacteroides*, and *Bifidobacterium* changed depending on the age. Interestingly, the categorization of young children fell into both “infant” and “adult” clusters despite their age, concluding that differences in the maturation process of humans also affect gut microbial composition.^15^

Variations in the human gut microbial maturation process begin at the time of conception. Mode of birth and infant feeding seem to affect gut colonization, confirming that the maternal gut microbiota is important for a newborn.^16^ Exhaustive studies have already been performed to characterize the human gut microbiota in individuals from 0 to 3 years old and in adulthood, but not in children from 3 to 18 years old due to difficulties obtaining samples or ethical/practical issues.^3^ Few or no studies can be found related to the gut microbiota of school-age children and their associations with MetS and T2DM; meanwhile, overweight and obesity belong to the most studied metabolic disorders in this population [Table 1 near here].

**Table 1.** Relevant gut microbiota studies conducted in pediatric subjects from different countries to unravel their relationship with metabolic disorders related with MetS

Country and age range	Number of study subjects	Health condition	Applied “omic” technologies	Relevant highest abundant bacterial communities	Reference
Switzerland 8–14 years old	30	Normal weight (NW) Obese (O)	16S rRNA gene metabarcoding Targeted metabolomics	No significant differences could be identified in individual microbial communities of NW and O subjects. Higher Bacteroidetes/Firmicutes ratio in NW compared to O.	^17^
Kazakhstan 7–13 years old	175	Normal weight (NW) Overweight (OW) Obese (O)	16S rRNA gene metabarcoding	Significant lower Bacteroidetes/Firmicutes ratio in O subjects compared to NW and OW individuals. Significant lower Bacteroidetes abundance in O subjects compared to NW individuals.	^18^
Italy 6–16 years old	78	Normal weight (NW) Obese (O)	16S rRNA gene metabarcoding Targeted metabolomics	Significant higher Firmicutes/Bacteroidetes ratio in O subjects compared to NW individuals. **Negatively associated with BMI**: Bacteroidetes phylum; *Bacteroides vulgatus* and *B. stercoris* **Positively associated with BMI**: Firmicutes phylum; *Faecalibacterium* prausnitzii **NW**: *Bacteroides* genus; *Bacteroides vulgatus* **O**: Firmicutes phylum	^19^
Mexico 9–11 years old	36	Undernourished (U) Normal weight (NW) Obese (O)	16S rRNA gene metabarcoding	Significant higher Firmicutes/Bacteroidetes ratio in U subjects compared to NW and O individuals. **U**: Firmicutes phylum; *Lachnospiraceae* family; *Prevotella* genus **NW**: Bacteroidetes phyum; *Bacteroides* genus **O**: Proteobacteria phylum	^20^
United States 7–18 years old	267	Underweight (UW) Normal weight (NW) Overweight (OW) Obese (O)	16S rRNA gene metabarcoding	**Negatively associated with BMI**: Actinobacteria phylum **Positively associated with BMI**: Proteobacteria phylum UW*: *Sutterella, Parabacteroides, Dorea, Serratia*, and *Pseudomonas* genera OW*: *Faecalibacterium, Serratia, Ruminococcus, Peptostreptococcaceae*, and *Coprococcus* genera O*: *Dialister, Acinetobacter, Bacillus, Bifidobacterium*, and *Lactobacillus* genera *Taking NW as reference	^21^
China 9–17 years old	58	Healthy (H) Obese with nonalcoholic liver disease (O-NAFLD) Obese non-NAFLD (O)	Shotgun metagenomics	**H**: Bacteroidetes phylum; *Lactobacillus, Oscillobacter*, and *Ruminiclostridium* genera O-NAFLD*: Proteobacteria phylum; Negativicutes class, *Phascolarctobacterium* genus, and *Phascolarctobacterium succinatuten;* γ-proteobacteria class, *Klebsiella* and *Kluyvera* genera, and *Klebsiella pneumoniae* and *Kluyvera ascorbata* species *Taking H as reference	^22^
Mexico 7–10 years old	27	Normal weight (NW) Obese (O) Obese with MetS (OMS)	16S rRNA gene metabarcoding Metatranscriptomics	**NW**: Bacteroidetes – Bacteroidia – Bacteroidales communities; *Phascolarctobacterium* genus **O**: *Porphyromonas* and *Faecalibacterium* genera (i.e. *Faecalibacterium prausnitzii); Bifidobacterium adolescents* **OMS**: Firmicutes and Proteobacteria phyla; *Colinsella, Catenibacterium* and *Coprococcus* genera; *Colinsella aerofaciens*	^23^
Spain 7–12 years old	39	Healthy (H) Obese with a 12-week training program (Oe) Obese (O)	16S rRNA gene metabarcoding Untargeted metabolomics	**H**: Actinobacteria phylum; Clostridia and Actinobacteria classes; *Clostridium, Bifidobacterium, Coprococcus, Akkermansia*, and *Streptococcus* genera **O**: Proteobacteria phylum (significantly decreased in Oe individuals); Bacteroidia, γ-proteobacteria (significantly decreased in Oe individuals), and β-proteobacteria classes; *Bacteroides, Prevotella, Phascolarctobacterium*, and *Paraprevotella* genera *Oe showed to start resembling more to H individuals	^24^
China 2–18 years old	51	Normal weight (NW) Obese (O)	16S rRNA gene metabarcoding	**NW**: Bacteroidetes phylum; *Oscillospira, Ruminococcus, Prevotella, Adlercreutzia, Sporobacter, Bifidobacterium, Clostridium, Desulfovibrio, Bilophila, Christensenella, Alistipes, Anaerotruncus, Eubacterium, Holdemania, Oxalobacter, Defluviitalea*, and *Collinsella* genera **O**: *Faecalibacterium, Turicibacter, Campylobacter, Actinobacillus, Aggregatibacter, SMB53, Rothia, Granulicatella, Streptococcus, Veillonella, Megamonas, Fusobacterium, Phascolarctobacterium, Haemophilus, Lachnospira*, and *Sutterella* genera	^25^
South Korea 7–18 years old	60	Normal weight (NW) Fat gain group (FGG) Fat loss group (FLG)	16S rRNA gene metabarcoding	**NW**: *Bacteroides, Oscillibacter*, and *Parabacteroides* genera **FGG**: Actinobacteria phylum; *Blautia, Dorea*, and *Fusicatenibacter* genera; *Eubacterium hallii* **FLG**: Clostridiales order and Clostridia class; *Blautia, Dorea*, and *Fusicatenibacter* genera; *Eubacterium hallii*	^26^

Studies in Dutch healthy children and adults demonstrated that the former possess a greater abundance of *Bifidobacterium* spp. and Bacteroidetes phylum (including the genera *Bacteroides* and *Prevotella*), while Firmicutes phylum was more predominant in adults (including the genera *Eubacterium, Clostridium, Dorea*, and *Coprococcus*), contradicting studies where a greater abundance of Firmicutes and lower Bacteroidetes levels were found in American healthy children aged from 7 to 12 years old, concluding that geographic origins may also impact gut microbiota-host interactions.^27,28^

Moreover, the pediatric gut microbiota has shown to be more flexible to environmental changes rather than that of adults, enriched with functions that may support host’s development meanwhile in adults possess more inflammatory, adiposity, and obesity functionality.^28^ Studies in Mexicans between 6 and 11 years old showed that the gut microbiota of obese individuals may resemble that in adults: obese children showed a higher abundance of Firmicutes and Actinobacteria and lower abundance of Bacteroidetes compared to normal weight children, which was similar to results obtained from Dutch adults.^27,29^ Interestingly, other studies revealed an increased richness and variability in gut microbial communities of obese Mexican children with a higher abundance of Firmicutes, Proteobacteria and Actinobacteria and a lower abundance of Bacteroidetes detected in obese individuals and obese with MetS individuals compared to normal weighted patients, similar to results obtained by Nirmalkar et al. (2018) in children from the same country.^23,29^

Apparently, it is not just lifestyle that can affect gut microbiota, but also aging, geographic region, and individual variability in the human microbial maturation process must be considered before designing gut microbiome–based interventions to treat or prevent MetS. Now, knowing that the gut microbiota of children is more flexible, additional efforts must be made to study this population which is prompt to respond positively and rapidly to microbial changes that may reverse deleterious medical conditions before developing irreversible diseases.

## Gut microbiota and its possible relationship with metabolic syndrome

**Figure 1.**
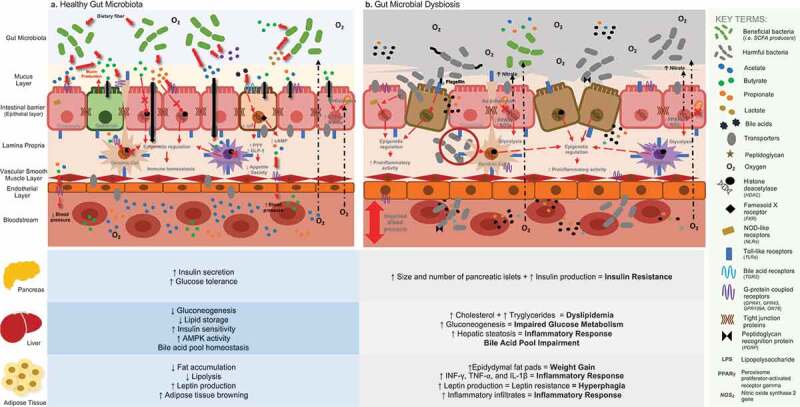
General overview of gut microbiota and its relationship with human metabolism according to recent findings retrieved from conventional techniques and “omic” technologies: **A**. The predominance of a hypoxic environment due to the presence of strict anaerobic bacteria and SCFA producers has been related with a healthy condition, promoting immune homeostasis, preserving the integrity of the intestinal barrier, and potentially being involved in the well-functioning of key organs and tissues that are relevant in the onset of MetS. **B**. Loss of the hypoxic environment due to an increased presence of facultative anaerobic bacterial communities and PAMP producers has been related to gut dysbiosis, increased intestinal permeability, and the triggering of proinflammatory activity, potentially causing a negative impact in the functionality of key organs and tissues that are involved in the development of MetS

Humans possess their own genome but also carry the gut microbiome, which affects their metabolism. Gut bacteria are involved in highly relevant activities that are crucial in the well-functioning of human’s metabolism [Figure 1a near here], but alterations in their composition may cause negative medical conditions. “Microbial dysbiosis” is the most popular term used to refer to these disparities, even though it has been recently cataloged as ambiguous due to the lack of a consensual definition within the scientific community and a poor characterization of how a healthy gut microbiota actually looks like.^30^ Mostly, it is used to refer to an increased abundance of harmful bacteria and a disturbance in beneficial microbial communities, which has shown to be possibly associated with obesity, hypertension, alterations in the nervous system, liver diseases, T2DM, and other maladies.^31^ For this reason, in the present review, this term and “microbial alterations” will be applied interchangeably.

During the last few years, “omic” technologies have gained popularity for the study of gut microbial functional potential, taxonomy, and human metabolism.^32^ The combination of metagenomics, transcriptomics, metabolomic, and proteomics has been useful for unraveling the relationship between microbiota and its host in multiple ways.^33,34^ However, conventional culture-based techniques are still needed as proof-of-concept, even though they can be labor-intensive and exclusively used with cultivable microorganisms.

Of note, the association of the gut microbiota with inflammation responses has shown to be highly relevant in the onset of MetS. Immune response and gut homeostasis seem to depend on gut microbiota–host bidirectional communication, which is mediated by the production and/or sensing of metabolites coming from the two involved.^35^ However, alterations in the microbial composition of the gut also seem to have an effect on the human metabolome, causing positive and/or negative effects on health and disrupting metabolic activities [Figure 1b near here]. Relevant bacterial products that have been related to MetS will be discussed herein.

### Short-chain fatty acids

Short-chain fatty acids (SCFAs) result from the fermentation of undigested carbohydrates and dietary fibers in the gastrointestinal tract. Acetate, propionate, and butyrate are the most abundant variants found in humans: acetate is the most abundant in the bloodstream, butyrate is mainly used by colonocytes as energy source, and propionate is mainly metabolized by the liver.^36,37^ They translocate into the circulatory system through carrier-mediated transporters and nonionic diffusion,^38^ helping to reach multiple organs and affect their functionality. Humans also possess free fatty acid (i.e., GPR43 and GPR41) and G protein-coupled receptors (i.e., GPR109A and OLFR78) to sense them in the gut epithelial layer, immune cells, pancreatic cells, adipose tissue, blood vessels, etc.^9,39^

These fatty acids provide protection, integrity, and energy to gut epithelial cells,^37^ but they can also regulate the production of peptide-YY (PYY) and glucagon like peptide-1 (GLP-1) in L-cells, gut hormones that regulate appetite, food intake, and glucose-induced insulin secretion and sensitivity.^40,41^ SCFAs also seem to modulate and maintain pressure homeostasis in blood vessels^42^ and regulate energy and glucose metabolism in adipose tissue and pancreatic cells, helping to reduce body weight, increase leptin levels, and improve insulin secretion.^37,43^

Moreover, they also have the ability to confer protection against pathogens and external threats, acting over immune cells.^39^ Butyrate and propionate inhibit histone deacetylases (HDACs), which are involved in DNA epigenetics, promoting anti-inflammatory (i.e. IL-18 and IL-10) and decreasing levels of proinflammatory cytokines (i.e. TNF-α, IFN-γ, and IL-6), maintaining homeostasis.^37,44^ The main SCFA producers belong to families of the Firmicutes phylum, but families from Fusobacteria, Actinobacteria, Thermotogue, and Spirochetes also seem to be butyrate producers. However, there are other communities belonging to *Bifidobacterium* and *Lactobacillus* genera that are not natural butyrate producers but can acquire this ability with the help of cross-feeding commensal bacteria producers of lactate and acetate, such as *Faecalibacterium, Eubacterium*, and *Roseburia*.^45^

Mitochondrial β-oxidation of butyrate is key for the host’s gut homeostasis, preserving its hypoxic environment.^46^ Studies in mice revealed that colonic epithelial hypoxia promotes the dominance of obligate anaerobes in the gut, such as SCFA producers, avoiding the increase in facultative anaerobes belonging to *Enterobacteriaceae* (i.e. *Salmonella enterica* serovar Typhimurium and *Escherichia coli*) which are microbial signatures of epithelial dysfunction.^47,48^ Thus, the lower abundance of SCFAs producers and increased facultative anaerobes may be a potential feature of gut dysbiosis. When gut microbial alterations take place, activation of the intestinal immune system occurs. A decrease in SCFA producers promote the gut’s microbial preference of less favorable substrates (i.e., mucins or dietary and endogenous proteins), causing an increase in intestinal permeability and the lower secretion of protective mucus,^45^ allowing the translocation of bacteria and harmful metabolites into the bloodstream.^48^

Metagenomic studies in the Chinese population showed a lower abundance of several butyrate-producing bacteria belonging to Firmicutes in prediabetic (Pre-DM) and treatment-naïve T2DM (TN-T2D) subjects and a higher abundance of *Enterobacteriaceae*, mostly dominated by *E. coli*. Also, a lower abundance of *A. muciniphila* and *C. bartlettii* was detected in TN-T2D compared to control and Pre-DM groups,^49^ concluding that hypoxic conditions may be relevant for gut homeostasis. *Akkermansia muciniphila* has previously been negatively correlated with overweight, obesity, untreated T2DM, and hypertension, while *Clostridium bartlettii* has been negatively associated with insulin resistance (IR).^50,51^ Of note, it is difficult to consider SCFAs as potential biomarkers of gut dysbiosis since 90–95% of these molecules produced in the colonic lumen are absorbed in the gut mucosa,^44^ making their quantification difficult in human samples; however, that does not mean that their study is not relevant to determine more about gut microbiota–host interactions.

### Pathogen-associated molecular patterns

Pathogen-associated molecular patterns (PAMPs) trigger responses in the host since the gut immune system responds to environmental changes and endogenous threats through the activation of pattern recognition receptors (PRRs). Toll-like receptors (TRLs) are a family of PRRs that sense not just a broad spectrum of microbial-associated (MAMPs) and damage-associated molecular patterns (DAMPs), but also PAMPs. TLR2, TLR4, and TLR5 interact the most with the human gut microbiota, since they are found in cellular membranes of gut epithelial and immune cells.^52,53^

Lipopolysaccharide (LPS) is a membrane component of gram-negative bacteria such as Bacteroidetes and Proteobacteria that induces chronic low-grade inflammation, promoting obesity and IR through the activation of TLRs, mainly TLR4.^54,55^ Studies found that mice fed a 4-week high-fat diet had increased levels of circulatory LPS, promoting inflammation in adipose tissue due to potential diabetogenic proinflammatory cytokines (i.e. IL-1, IL-6 and TNF-α), weight gain, and liver IR.^56^

Moreover, flagellin is another component that activates TLRs (mainly TLR5) and induces the production of proinflammatory cytokines (i.e. TNF-α, IL-6, and IL-1β).^52^ Increased levels of flagellated bacteria, such as communities belonging to γ-Proteobacteria (i.e. motile pathobiont *E. coli* strains) and Firmicutes, have been correlated with multiple inflammatory diseases where MetS is involved, since they upregulate motility-related gene expression that boosts gut microbial production of flagellin, which increases microbiota encroachment into the mucus layer and proinflammatory potential within the host.^57^

Metagenomic studies in normal-weighted Mexican children (NW), obese (O), and obese with MetS (OMS) from 7 to 10 years old revealed the higher abundance of Firmicutes and lower abundance of Bacteroidetes in O and OMS compared with NW children. Furthermore, OMS subjects seem to possess a higher abundance of Proteobacteria. Interestingly, introducing metatranscriptomics to this work helped to detect a significant prevalence of membrane and surface proteins in the transcriptome of these children, suggesting that bacterial components can be related to the onset of chronic inflammation that is responsible for metabolic disorders.^23^ Something similar was seen in Chinese adults, where a higher abundance of unique Proteobacteria (mainly from *Escherichia, Citrobacter*, and *Enterobacter*) was detected in prediabetic subjects. In this study, coupling metaproteomic approaches also helped to reveal that 90% of the genes and meta-proteins found were assigned to Firmicutes, Bacteroides, and Proteobacteria, which have already been seen to be the main producers of LPS and flagellins.^49^ This is how both studies show the relevance of bacterial membrane components in the study of inflammatory diseases, with several of them having been confirmed to trigger host immune responses and their abundance being dependent on the microbial composition of the gut.

Finally, peptidoglycan (PG) is a PAMP that is present in the outer membrane of gram-positive and gram-negative bacteria. It can be recognized by TLRs (mainly TLR2), secreted PG recognition proteins (PGLRPs) produced by the host, and nucleotide-binding oligomerization domain (NOD)-like receptors (NLRs). Some NLRs possess the ability to create “inflammasomes” that trigger proinflammatory cytokines (i.e. IL-1β and IL-18) related to the pathogenesis of inflammation in cases of obesity and IR.^58,59^ Recent studies have shown associations between PG and MetS in adult *Drosophila* models, where a decrease in insulin signaling was observed due to the activation of PG-dependent NF-kB, together with alterations in gut microbial communities;^58^ however, this still needs to be confirmed in humans.

### Bile acids

Bile acids (BAs) are products from hepatic cholesterol metabolism that help to absorb dietary lipids and vitamins.^60^ Primary bile acids (PBAs) and secondary bile acids (SBAs) are also involved in the activation of intestinal nuclear farnesoid X receptor (FXR) which has a crosstalk interaction with G protein-coupled bile acid receptor (TGR5).^61,62^ FXR is more involved in GLP-1 secretion, helping to improve glucose and insulin sensitivity, while TGR5 is more focused on the browning process, energy metabolism, and decreasing obesity in adipose tissue.^63^

The gut microbiota seems to be involved in BA metabolism since the deconjugation of PBAs in the small intestine is performed by bacterial bile salt hydrolases (BSH) from a wide variety of gram-positive (i.e., *Bifidobacterium, Clostridium, Lactobacillus, Enterococcus*, and *Acetatifactor*) and gram-negative bacteria (i.e., *Bacteroides acidifaciens*). Moreover, bacterial hydroxysteroid dehydrogenase (HSDH) from specific bacteria belonging to *Bacteroides, Clostridium, Lactobacillus, Eubacterium*, and *Escherichia* genera convert PBAs into SBAs, where cholic acid (CA) is converted into lithocholic acid (LCA) and chenodeoxycholic acid (CDCA) into deoxycholic acid (DCA) for their reabsorption and return to the liver.^64^ During the bacterial processing of PBAs, BAs also exert antimicrobial properties due to their detergent effect on bacterial cell membranes, inducers of DNA damage, and disruptors of protein structures that may also affect gut microbiota.^65^

Alterations in the BA pool have been related to colon cancer, inflammatory bowel disease, cholestatic liver disease, and MetS. When gut microbial alterations occur, the BA pool becomes dysregulated, acquiring an increased proinflammatory and cytotoxic potential that may affect the liver.^62^ Metagenomic studies using mice cecal content showed that the abundance of *Acetatifactor*, belonging to the *Clostridium* cluster XIV, and *Bacteroides* depends on the activation of FXR. Both bacterial communities possess high BSH and 7α- and 7β-dehydroxylase activity that help in the production of LCA, which is involved in the activation of TGR5 signaling.^65^

Metagenomic studies in Italian NW and O children showed an increased depletion of *Bacteroides* spp. in O subjects and a negative correlation with BMI z-score, supporting the idea that this genus is relevant in bile acid homeostasis.^19^ Furthermore, metagenomic and metabolomic studies in Japanese adults proved what was previously found in mice: *Clostridium* cluster XIVa (i.e. *C. scindens, C. hylemonae, Ruminococcus gnavus*, and *Peptostreptococcus productus*) was lower in subjects with inflammatory diseases, specifically with Crohn’s disease and ulcerative colitis, compared to the control group and measurements of BAs in serum and feces showed that this cluster was probably the higher contributor of 7α-dehydroxylation of BAs to produce SBAs (LCA and DCA) and significant in 7β-dehydrogenation of BAs.^66^

All of the above-mentioned facts highlight the relevance of FXR-TGR5 crosstalk mediated by the BA pool in the shaping of the gut microbiota and the importance of SBA producers in bile acid metabolism, glucose, and energy homeostasis. However, further research needs to be performed to understand the role of BAs in MetS, and it is necessary to study the effect of BA metabolism in the gut microbiota of children and compare it with that of adults. It has already been reported that the alternative or “acidic” pathway to produce PBAs in the liver seems to be preferred by human metabolism during childhood compared to the classic or “neutral” pathway that is more common in adults,^64^ something that may affect the maturation process of gut microbiota through life.

### Colonic gases

Hydrogen (H_2_) and carbon dioxide (CO_2_) are the major byproducts of microbial anaerobic fermentation, which can be released to the intestinal environment or taken by other bacteria.^62^ For example, H_2_ can be used by bacteria to produce sulfide, acetate, and methane. The main bacterial producers of H_2_ belong to the Firmicutes and Bacteroidetes phyla (i.e., *Roseburia, Ruminococcus, Bacteroides, Clostridum*, and *Eubacterium*) and CO_2_ is produced by bacteria such as *Clostridium* species (i.e., *C. sporogenes, C. butyricum*, and *C. perfringens*), which also have the ability to produce both gases.^65,67^

Methanogens, which are mainly composed of archaea, use both gases to produce methane (CH_4_). *Methanobrevibacter smithii* is the only methanogen known to live within the human gut, but *Methanosphaera stadtmanae* can also sometimes be observed.^68^ *M. smithii* has been related to increased BMI and impaired glucose intolerance in children and adults, respectively, but studies are still inconclusive since others report lower levels of this archaea in obese subjects.^69–71^

Sulfate-reducing bacteria also use H_2_ to produce hydrogen sulfide (H_2_S). *Desulfovibrio* spp. is the most highly active species and greatest affinity bacteria for H_2._ Since both sulfate‐reducing bacteria and methanogens from the gut compete for the same pool of H_2_, the availability of sulfate for H_2_S production is the bottleneck for this preference.^65,67^ *Desulfovibrio piger* has been associated with abundance of *Collinsella aerofaciens*, an Actinobacterium that conducts sugar fermentation removing H_2_, lactate, and formate. This is relevant since *C. aerofaciens* has been linked with BA metabolism, the regulation of blood cholesterol, the production of SCFAs, and gut homeostasis.^72,73^ However, production of H_2_S in the gastrointestinal tract seem to also be related in arterial blood pressure homeostasis and provide cardioprotective effects, but mechanisms are still unclear.^72^ Of note, a higher concentration of H_2_S seems to be relevant for liver and adipose tissue since it has been shown to promote the regulation of insulin sensitivity, stimulate hepatic gluconeogenesis and glycogenolysis, inhibit glucose and glycogen storage usage, and regulate lipolysis, inflammation, and adipokine production.^74^

Finally, acetogenesis also requires H_2_ as a substrate, contributing positively to energy harvest. Firmicutes phylum has been shown to be the largest group of characterized gut acetogens in several mammals.^67,68^ *Blautia hydrogenotrophica* and *Ruminococcus bromii* are some examples of bacteria involved in formate uptake to produce acetate.^75^ Metagenomics coupled with metabolomic and transcriptomic approaches concluded that human obese subjects with IR possessed a lower relative abundance of Firmicutes, while Bacteroidetes and Proteobacteria were increased compared to insulin-sensitive individuals. Firmicutes, specifically belonging to the *Ruminococcaceae* family, were positively correlated with subcutaneous adipose tissue, plasma acetate, and insulin sensitivity in brown adipocytes,^76^ inferring their relevance in obesity and MetS. Moreover, studies in Mexican children showed that *Ruminococcaceae* UCG-002 was present in normal weighted individuals and appeared to be negatively associated with obesity but positively associated with fasting plasma insulin, highlighting the relevance of studying the role of the gut microbiota in human intermediate metabolism.^77^

### Branched-chain and aromatic amino acids

Branched-chain amino acids (BCAAs) have been associated with metabolic features related to MetS, the onset of T2DM, and CVDs. They are composed of leucine, isoleucine, and valine, which cannot be synthesized by humans but can be obtained from exogenous sources such as the diet.^78^ It is believed that increased circulatory BCAA levels may be involved in the accumulation of incompletely oxidized fatty acids and glucose, impaired response to insulin, alterations in glucose homeostasis, and mitochondrial stress.^78,79^

Metabolomic and metagenomic studies in Danish adults found bacterial species with an enriched potential of BCAA biosynthesis and decreased BCAA bacterial inward transportation. *Prevotella copri* and *Bacteroides vulgatus* were the main bacterial species associated with IR, while *Butyrivibrio crossotum* and *Eubacterium siraeum* were negatively linked to this condition. Forty-one microbial functional modules were detected and correlated with IR and MetS phenotypes, mostly belonging to vitamin and cofactor biosynthesis, and BCAA, LPS, and transport systems. Methanogenesis, the inward transport of BCAAs, pyruvate oxidation and transport systems were more negatively associated with IR and Homeostatic Model Assessment for IR (HOMA-IR).^80^

Moreover, metabolomic approaches in children detected high BCAA levels in Austrian subjects from 9 to 19 years old diagnosed with severe obesity, being positively correlated with liver fat content. Interestingly, liver fat content was also positively correlated with acylcarnitines C3-, C4-, and C5- which are BCAA degradation by-products, confirming the potential role of BCAAs in metabolic disparities.^81^

On the other hand, aromatic amino acids (AAAs) such as tryptophan, phenylalanine and tyrosine have also been correlated with IR, T2DM, and CVDs. Studies in Chinese obese subjects showed high levels of circulatory BCAAs and AAAs coupled with the higher gut microbial capacity for producing them. AAAs showed a positive correlation with HOMA-IR, hyperlipidemia, inflammatory circulatory factors, and hyperglycemia. Additionally, *B. thetaiotaomicron* and *B. ovatus* were found to take and ferment AAAs, concluding that a depletion of *Bacteroides* spp. in obese subjects may potentially cause increased levels of AAAs.^82^

Additionally, the production of deleterious microbial metabolites has also been related to several AAAs. Phenylalanine and tyrosine have been reported to be taken by certain gut microbial communities to produce phenolic end compounds such as phenol and *p*-cresol previously linked with cytotoxicity, genotoxicity, promoters of paracellular permeability and the reduction of endothelial barrier function *in vitro*, etc. Bacteria belonging to *Enterococcaceae, Clostridiaceae, Staphylococcaceae*, and *Enterobacteriaceae* have been shown to be predominant phenol-producers, while *p*-cresol has been found to be produced by certain *Clostridium* species, *Bacteroidaceae, Bifidobacteriaceae, Eubacteriaceae, Lachnospiraceae, Porphyromonadaceae, Ruminococcaceae, Veillonellaceae* and *Fusobacteriaceae*.^83,84^

Finally, in a 7.5-year longitudinal study from childhood to early adulthood in nondiabetic Finnish girls, serum concentrations of BCAAs and AAAs showed the strongest correlation with HOMA-IR. They concluded that IR starts to develop in early puberty and reached its peak in mid-puberty probably due to high metabolic and hormonal changes.^85^ Gut microbiota was not analyzed, but it denotes the significance of researching these molecules in school-age children since gut microbial maturation may also be influenced by metabolic and hormonal alterations that are characteristic of puberty.

## Gut microbiome-based interventions for the prevention and treatment of disorders related to metabolic syndrome

Prebiotics are gaining popularity since they serve as food for beneficial gut microbes, having a positive impact on human health. Studies in randomly selected obese, overweight, and healthy Canadian children from 7 to 12 years old showed the role of oligofructose-enriched inulin (OI) in the behavior and composition of the gut microbiota. After 16 week-consumption, subjects showed a significant reduction in body weight, body fat, and trunk fat. Moreover, they showed a relevant reduction of IL-6 levels and serum triglycerides compared to the placebo group. In addition, *Bifidobacterium* spp. (linked with normal-weight conditions) was increased and *Bacteroides vulgatus* (linked to IR) was decreased compared with the control group.^86^

The discovery, characterization, and use of microorganisms that seem to be associated with health have also recently gained interest; for example, probiotics, which have already demonstrated interesting benefits in school-age children. A study conducted in Malaysian children from 7 to 10 years old with NW and O conditions that consumed a fermented probiotic drink enriched with *Lactobacillus casei* Shirota for 4 weeks showed significant increases in *Lactobacillus* and *Bifidobacterium* abundance compared to in the control groups. Additionally, fecal SCFA levels were altered since total SCFAs and propionic acid had a significant increase in NW and O after consuming the beverage compared to levels reported at the beginning of the intervention.^87^ Moreover, studies in Japanese children from 5 to 10 years old given a probiotic drink enriched with the same microorganism had similar results after 6 months of consumption. They had an increase in *Lactobacillus* and *Bifidobacterium* but also a significant decrease in *Enterobacteriaceae, Staphylococcus* and *Clostridium perfringens* communities. However, these changes appeared to cease 6 months later when individuals arrested its consumption, concluding that the regular use of probiotics may help in the restoration of the gut microbiota.^88^

Furthermore, dietary assessment may be helpful in the evaluation of diet- and nutrition-associated variations and their impact on gut microbial abundance and variability. A research in Filipino pediatric subjects with NW and O conditions was conducted to determine how differences in macronutrients, dietary fiber, and total energy intakes affect their gut environment and microbial communities. In the end, there were no significant differences in these dietetic components in their gut microbiota diversity and structure, since both study groups were shown to follow a mixture of traditional and Western nutritional patterns, deriving inconclusive results. However, the relative abundance of *Bifidobacterium, Turicibacter* and *Clostridiaceae* 1 was statistically higher in NW individuals compared to O subjects, and *Lachnospira, Erysipelotrichaceae* UCG-003 and *Peptostreptococcaceae* genera were statistically higher in O subjects with higher dietary fiber consumption compared to those who consume it in lower quantities.^89^ Despite these inconclusive results, dietary assessment may be promising if inclusion/exclusion criteria are well established to work with more standardized study groups.

Finally, it is important to note that there have been no studies conducted within pediatric population to treat or prevent MetS, but they scarcely exist in adult subjects. A study reported in overweight/obese insulin-resistant adults supplemented with pasteurized *Akkermansia muciniphila* for 3 months showed a significant improvement in insulin sensitivity, and a decrease in plasma total cholesterol and insulinemia compared to the placebo group, with no significant alterations in gut microbiome structure. Moreover, it also showed a decrease in body weight, fat mass content, and hip circumference, but with no statistical significance.^50^

## Challenges and future perspectives

Studying pediatric subjects is a hard task due to the complexity of retrieving samples and considering variations in their hormonal and gut microbial maturation processes since the time of conception. However, knowing more about gut microbial flexibility and apparent enriched functionality that supports the host’s development may help to reveal interesting insights regarding gut microbiota and its possible association with MetS from the early stages of life. “Omic” technologies have helped during the last years to study gut microbiome–host interactions with the use of noninvasive biological samples but they cannot completely replace conventional techniques conducted *in vitro* and/or *in vivo* using animal models since they are still needed as proof-of-concept.

Some studies have shown that the gut microbiota of obese children may resemble that in adults, but this needs to be studied in more detail. Variations among both groups are important since diet, lifestyle, and aging have been shown to probably be involved in the shaping of the gut microbiota. Of note, there are other unknown and known features, such as genetics, drug consumption, and demographic background that may also be involved in gut microbial alterations, adding greater difficulty to its study. Moreover, increasing population-based cohort studies on gut microbiota composition found in countries with high rates of childhood obesity and MetS may also help to learn more about these microbial communities and their role in human metabolism at different stages of life, with greater accuracy, less variability, and the possibility of considering hormonal fluctuations that could affect these interactions.

Furthermore, interactions, even among microbial communities, are barely studied and must be considered in future research, since viruses, archaea, and fungi also inhabit the human gut. For all of the aforementioned, it is concluded that there is still a lot of work to do regarding the role of gut microbiota in MetS and other cardiometabolic conditions, but mysteries must be unraveled one step at a time to develop future and effective microbiome-based interventions that may help to improve the quality of life of children and adolescents that are now exposed to the increasing prevalence of obesity and MetS. The gut microbiota-brain axis was not reviewed here, but it should also not be disregarded since it has been shown to have an important role in human mechanisms of disease.

## Acronyms

AAA – aromatic amino acid; BA – bile acid; BMI – body mass index; BCAA – branched-chain amino acid; BSH – bile salt hydrolase; CO_2_ – carbon dioxide; FXR – nuclear farnesoid X receptor; H_2_ – hydrogen; H_2_S – hydrogen sulfide; HOMA-IR – homeostatic model assessment for insulin resistance; IR – insulin resistance; LPS – lipopolysaccharide; MetS – metabolic syndrome; PAMP – Pathogen-associated molecular pattern; PBA – primary bile acid; PG – peptidoglycan; NOD – nucleotide-binding oligomerization domain; NLR – NOD-like receptor; NW – normal weight; O – obese; SBA – secondary bile acid; SCFA – short-chain fatty acid; TGR5 – G protein-coupled bile acid receptor; TLR – toll-like receptor; T2DM – type-2 diabetes mellitus.
